# Supplementary material for: A type-specific B-cell epitope at the apex of outer surface protein C (OspC) of the Lyme disease spirochete, Borreliella burgdorferi
Source: Microbiol Spectr. 2025 Feb 14;13(4):e02883-24. doi: 10.1128/spectrum.02883-24 (PMC11960070; doi:10.1128/spectrum.02883-24)
Supplement: Supplemental figures — Fig. S1 to S3. [file spectrum.02883-24-s0001.pdf]

Figure S1

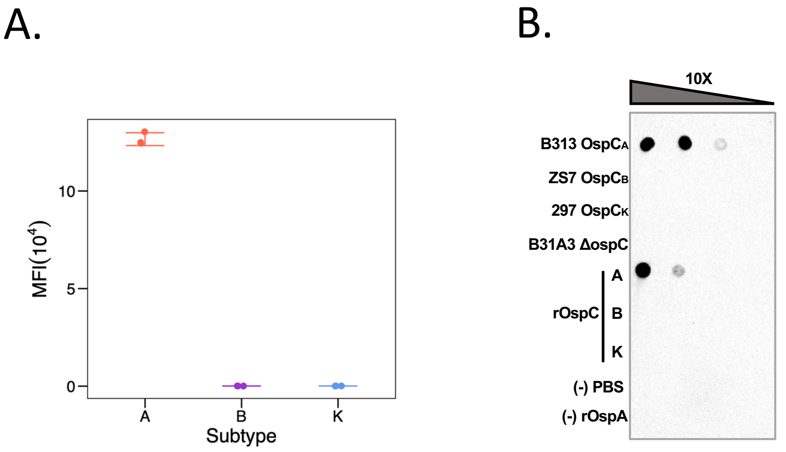

Fig S1. 8C1 hybridoma supernatant is OspC Type A specific. A) Hybridoma supernatant recognizes rOspC Type A but not B and K in Luminex. B) Dot blot analysis where $1x10^7$ bacteria or 1 µg of recombinant OspC protein types A, B, and K, were diluted and spotted on a nitrocellulose membrane and incubated with mouse hybridoma supernatant containing 8C1 IgG. The membrane was probed with an HRP-labelled anti-mouse IgG secondary antibody for detection of IgG binding. PBS and an unrelated protein, OspA were diluted and spotted as controls.

Figure S2

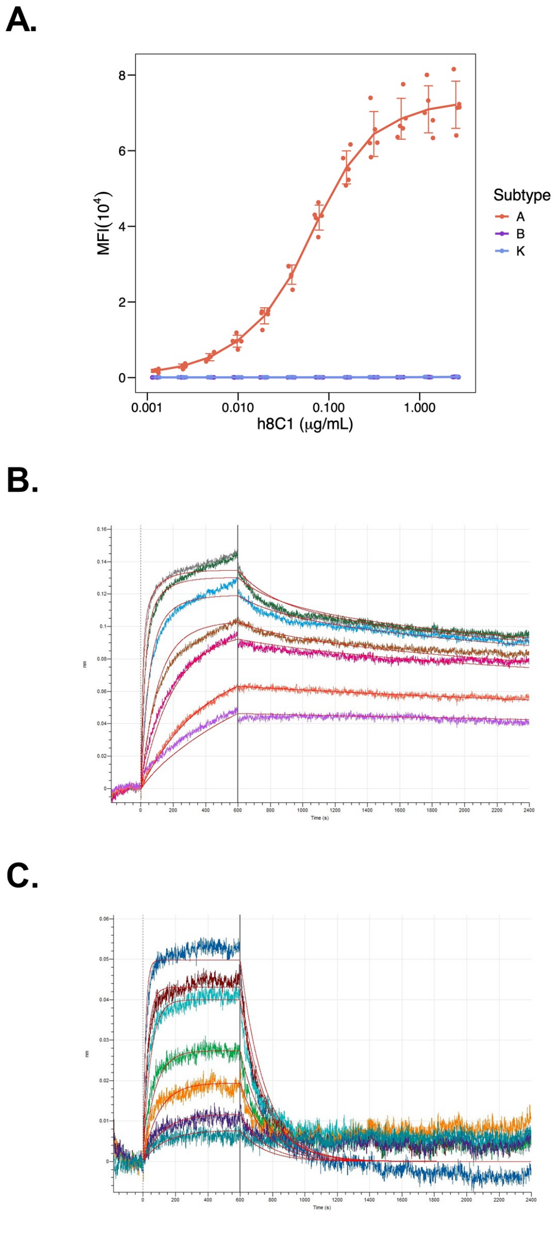

Fig S2. Characterization of recombinant purified 8C1. A) Recombinant OspC Types A, B and K were coupled to Luminex beads, and then incubated with a dilution series of purified humanized 8C1. 8C1 displayed a clear dose response binding curve against $rOspC_A$ but did not recognize $OspC_B$ or $OspC_K$ at any concentration tested. B-C) Biolayer interferometry sensorgrams of 8C1 mAb (B) or Fab (C) binding to $OspC_A$.

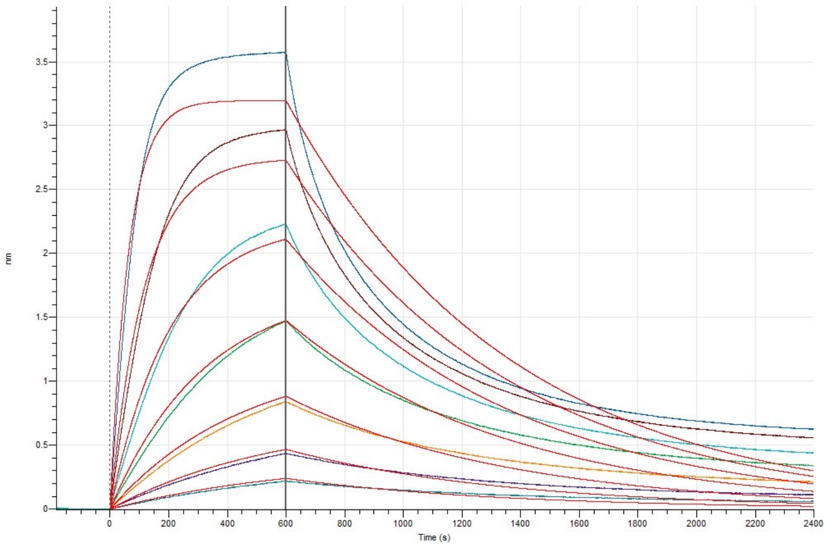

Fig S3. 8C1 Fabs binding to $OspC_A$-derived peptide 130-150. Biolayer interferometry sensorgrams of Fabs of 8C1 binding to a peptide composed of residues 130-150 from $OspC_A$. The data is fit with a 1:1 binding model and gives a K_D of 9.2 nM.
